# Efficacy and safety of oral and sublingual versus vaginal misoprostol for induction of labour: a systematic review and meta-analysis

**DOI:** 10.1007/s00404-022-06867-9

**Published:** 2022-12-06

**Authors:** Vasilios Pergialiotis, Michail Panagiotopoulos, Therapon Constantinou, Lito Vogiatzi Vokotopoulou, Andreas Koumenis, Sofoklis Stavros, Andreas Voskos, George Daskalakis

**Affiliations:** 1 1st Department of Obstetrics and Gynecology, Alexandra Hospital, National and Kapodistrian University of Athens, Lourou 2-4, 11528 Athens, Greece; 2 Laboratory of Experimental Surgery and Surgical Research N.S. Christeas, Athens, Greece

**Keywords:** Misoprostol, Oral, Sublingual, Induction of labour, Maternal morbidity, Neonatal morbidity, Systematic review, Meta-analysis

## Abstract

**Objective:**

Misoprostol is a synthetic PGE_1_ analogue that is used for induction of labour. Current guidelines support the use of doses that do not exceed 25 mcg in order to limit maternal and neonatal adverse outcomes. The present meta-analysis investigates the efficacy and safety of oral compared to vaginally inserted misoprostol in terms of induction of labor and adverse peripartum outcomes.

**Methods:**

We searched Medline, Scopus, the Cochrane Central Register of Controlled Trials CENTRAL, Google Scholar, and Clinicaltrials.gov databases from inception till April 2022. Randomized controlled trials that assessed the efficacy of oral misoprostol (per os or sublingual) compared to vaginally inserted misoprostol. Effect sizes were calculated in R. Sensitivity analysis was performed to evaluate the possibility of small study effects, p-hacking. Meta-regression and subgroup analysis according to the dose of misoprostol was also investigated. The methodological quality of the included studies was assessed by two independent reviewers using the risk of bias 2 tool. Quality of evidence for primary outcomes was evaluated under the Grading of Recommendations Assessment, Development and Evaluation (GRADE) framework, ranging from very low to high.

**Results:**

Overall, 57 studies were included that involved 10,975 parturient. Their risk of bias ranged between low-moderate. There were no differences among the routes of intake in terms of successful vaginal delivery within 24 h (RR 0.90, 95% CI 0.80) and cesarean section rates (RR 0.92, 95% CI 0.82, 1.04). Sublingual misoprostol was superior compared to vaginal misoprostol in reducing the interval from induction to delivery (MD – 1.11 h, 95% CI – 2.06, – 0.17). On the other hand, per os misoprostol was inferior compared to vaginal misoprostol in terms of this outcome (MD 3.45 h, 95% CI 1.85, 5.06). Maternal and neonatal morbidity was not affected by the route or dose of misoprostol.

**Conclusion:**

The findings of our study suggest that oral misoprostol intake is equally safe to vaginal misoprostol in terms of inducing labor at term. Sublingual intake seems to outperform the per os and vaginal routes without increasing the accompanying morbidity. Increasing the dose of misoprostol does not seem to increase its efficacy.

**Clinical trial registration:**

Open Science Framework (https://doi.org/10.17605/OSF.IO/V9JHF).

## What does this study add to the clinical work

**Table:** 

Current evidence indicates that oral (per os and sublingual) misoprostol intake is equal to vaginal misoprostol in terms of inducing labor as well as associated maternal and neonatal morbidity.	

## Introduction

Induction of labour is used for decades in obstetrics; however, during the last years a steep rise in the percentage of deliveries that are medically induced is observed which from anecdotal reports seems to reach approximately 9.6% of all deliveries worldwide [1]. In the United States these rates are considerably higher reaching approximately 27%, while in Europe they seem to range between 6.8 and 33% [2, 3]. Whereas several methods have been used to induce cervical ripening as well as onset of labor, including oxytocin, prostaglandins, laminaria tents and foley balloon catheter, prostaglandins and oxytocin are the most widely accepted.

The American College of Obstetricians and Gynecologists suggests the use of prostaglandins for cervical ripening and labor induction at intervals that should be at least 3–6 h apart to avoid the risk of uterine tachysystole, although the minimum interval that is considered safe has not been standardized yet [4]. Regarding misoprostol, the World Health Organization recommends either oral (25 μg, 2-hourly) or vaginal route (25 μg, 6-hourly) for induction of labour [1]. In a previous meta-analysis that was conducted by Alfirevic et al., misoprostol combined with oxytocin and amniotomy has been proven to be the best method for achieving vaginal delivery within 24 h [5].

Misoprostol was originally licensed for oral use; however, vaginal and sublingual routes of administration are becoming more and more popular supported by pharmacokinetic studies focusing on the systemic bioavailability parameters achieved [6]. While the sublingual route seems to have the greater bioavailability, safety concerns have been raised from studies that were conducted in first trimester terminations [7].

Several articles have addressed the efficacy of the various routes of misoprostol in inducing a successful vaginal delivery and a previous meta-analysis that was published in 2008 suggested that both sublingual and vaginal routes seem to be comparable in terms of achieving vaginal delivery [8]. More recently, Alfirevic et al. published a systematic review comparing oral misoprostol to other methods of induction of labor (including oxytocin) and observed that it was associated with fewer cesarean sections [9]. Since then, several randomized trials have been published and an update is indicated to review current knowledge as, to date, there is no consensus on the optimal route of misoprostol intake for induction of labour.

The purpose of this meta-analysis is to investigate whether oral (either per os or sublingual) misoprostol intake is superior to vaginal administration in terms of inducing labor and leading to a vaginal delivery. Taking in mind the safety concerns that have been raised in the field of first trimester pregnancy complications, we also investigated its impact on maternal and neonatal morbidity outcomes.

## Materials and methods

### Protocol and registration

The present meta-analysis was designed according to the Preferred Reporting Items for Systematic Reviews and Meta-Analyses (PRISMA) guidelines [10]. The study was based in aggregated data that have been already published in the international literature. Patient consent and institutional review board approval were not retrieved as they are not required in this type of studies. The study's protocol was published in open science framework, prior to the conduct of this review (Registration https://doi.org/10.17605/OSF.IO/V9JHF).

### Eligibility criteria

Eligibility criteria for the inclusion of studies were predetermined. Randomized trials that compared the efficacy of oral (both per os or sublingual) to that of vaginal misoprostol in terms of induction of labour were considered eligible for inclusion. Quasi-randomized trials as well as observational studies (prospective and retrospective studies) were omitted from the systematic review. Only studies investigating induction of labor outcomes in singleton vertex presentations were included. Studies were included irrespective of the actual reason for induction of labour (antenatal pathology or prolonged pregnancy).

### Information sources and search strategy

Two authors (V.P and M.P.) searched Medline (1966–2021), Scopus (2004–2021), Clinicaltrials.gov (2008–2021), EMBASE (1980–2021), Cochrane Central Register of Controlled Trials CENTRAL (1999–2021) and Google Scholar (2004–2021) along with the reference lists of electronically retrieved full-text papers. The date of the last search was set at April 30, 2022. No date restrictions were applied. Articles were limited to English language. The search strategy included the text words “induction; labour; misoprostol; oral; vaginal; sublingual; per os; oral” and is presented in brief in “Appendix”.

Studies were selected in three consecutive stages. Following deduplication, the titles and abstracts of all electronic articles were independently screened by three authors (V.P., M.P and T.K.) to assess their eligibility. The decision for inclusion of studies in the present meta-analysis was taken after retrieving and reviewing the full version of articles that were considered potentially eligible. Discrepancies that arose in this latter stage were resolved by consensus from all authors.

### Study selection and data extraction

Outcome measures were predefined during the design of the present systematic review. Data extraction was performed using a modified data form that was based in Cochrane`s data collection form for intervention reviews for RCTs and non-RCTs [11].

The main outcomes of our study were the rates of successful vaginal delivery within 24 h, as well as cesarean section rates. Secondary outcomes included successful vaginal delivery beyond the limit of 24 h, interval to delivery, risk of uterine tachysystole, risk of postpartum hemorrhage and its accompanying side effects (risk of disseminated intravascular coagulation and need for obstetric hysterectomy) and neonatal side effects (including Apgar score values at 5` and need for admission to the NICU).

### Assessment of risk of bias and quality of evidence

The methodological quality of the included studies was assessed by two independent reviewers using the risk of bias 2 (RoB 2) tool. RoB2 incorporates five domains that include assessment of (i) risk of bias that arises from the randomization process, (ii) risk of bias due to deviations from the intended interventions, (iii) missing outcome data, (iv) risk of bias in the measurement of the outcome, and (v) risk of bias in the selection of the reported result.

Quality of evidence was evaluated under the Grading of Recommendations Assessment, Development and Evaluation (GRADE) framework, ranging from very low to high. More specifically, credibility of evidence will be assessed by taking into account the following domains: study limitations, directness, consistency, precision and publication bias. In particular, study limitations were evaluated based on risk of bias assessments (RoB 2 score), while directness was judged using the PICOS (population, intervention, comparison, outcome, study type) approach.

### Synthesis of results

Statistical meta-analysis was performed with RStudio using the *meta and metafor* functions (RStudio Team (2015). RStudio: Integrated Development for R. RStudio, Inc., Boston, MA URL http://www.rstudio.com/). Statistical heterogeneity was not considered during the evaluation of the appropriate model of statistical analysis as the anticipated methodological heterogeneity of included studies did not leave space for assumption of comparable effect sizes among studies included in the meta-analysis [12]. Confidence intervals were set at 95%. We calculated pooled mean differences (MD) and 95% confidence intervals (CI) with the Hartung-Knapp-Sidik-Jonkman instead of the traditional Dersimonian-Laird random effects model analysis (REM). The decision to proceed with this type of analysis was taken after taking into consideration recent reports that support its superiority compared to the Dersimonian-Laird model when comparing studies of varying sample sizes and between-study heterogeneity [13]. When variables where expressed as median (range), median (interquartile range) or interquartile range and sample size transformation where performed to acquire the mean and standard deviation to include the studies in the meta-analysis [14].

Publication bias was assessed by examining the possibility of small-study effects through the visual inspection of funnel plots. The asymmetry of funnel plots was statistically evaluated using the Egger’s regression and Begg-Mazumdar’s rank correlation tests. The Duval and Tweedie`s trim and fill method was applied to impute missing effects irrespective of the asymmetry of the funnel plot.

Publication bias was evaluated by examining the potential presence of small-study effects through the visual inspection of funnel plots. Rücker’s Limit Meta-Analysis was applied to account for bias that arises due to small-study effects for primary outcomes. Outlier analysis was also undertaken to evaluate the effect of outlier studies on the overall effect size.

#### Prediction intervals

Prediction intervals (PI) were also calculated, using the *meta* function in RStudio, to evaluate the estimated effect that is expected to be seen by future studies in the field. The estimation of prediction intervals takes into account the inter-study variation of the results and express the existing heterogeneity at the same scale as the examined outcome.

#### Subgroup analysis

Subgroup analysis was designed following the retrieval of studies as several articles evaluated differences among sublingual and vaginal misoprostol as well as between per os and vaginal misoprostol; hence, oral misoprostol was subgrouped (as per os and sublingual) to evaluate for differences in summary effect estimates among the two methods.

Given the fact that in their previous meta-analysis Alfirevic et al. commented the lack of a substantial number of studies that could permit subgroup analysis based on the dose of misoprostol [9] in the present meta-analysis we performed meta-regression analysis of primary and secondary outcomes based on differences in misoprostol dosage (per dose administered and not total dose) among the two groups, to evaluate the potential effect of differences in the effect estimate. Subgroup analyses were also performed after arbitrary grouping of studies according to the maximum dose administered (Low dose = both groups received a dose of < 50 mcg of misoprostol, Intermediate = at least one of the two groups received = 50 < 100 mcg of misoprostol, High dose = at least one of the two groups received ≥ 100 mcg of misoprostol).

## Results

### Study selection and study characteristics

Following completion of the electronic search strategy we were able to identify 1436 potentially relevant articles. After reading the abstracts and, when needed, full texts we managed to limit them to an overall number of 69 articles of which we finally selected 57 randomized trials that involved 10,975 parturient [15–70]. The methodological characteristics of included studies as well as patient characteristics are depicted in the “Appendix” and reveal comparable groups of oral vs vaginal misoprostol intake in terms of maternal age and body mass index, gestational age at delivery parity, Bishop score prior to the start of induction and neonatal birthweight.

### Risk of bias of included studies

**Fig. 1:**
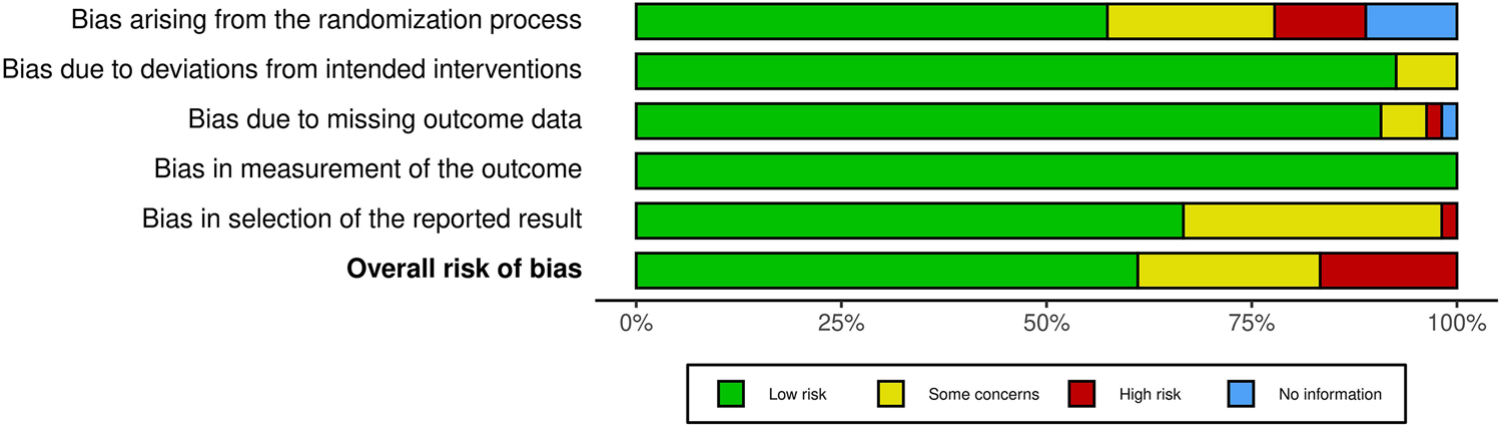
Summary risk of bias 2 (RoB 2) plot

The evaluation of the methodological quality of included studies with the RoB2 tool revealed low risk of bias for the majority of studies, whereas some concerns were raised in 12 studies and high risk of bias was revealed in 9 studies (Fig. 1 and “Appendix”).

### Synthesis of results

Overall, effect sizes of outcomes regarding per os and sublingual misoprostol compared to vaginal are presented in Table 1, while summary effect sizes along with subgroup analysis according to maximum misoprostol dose are presented in Table 2.

**Table 1:** Effect sizes of outcomes regarding per os and sublingual misoprostol compared to vaginal

Outcome	RR/MD (95% CI)	Number of studies
Per os misoprostol compared to vaginal		
Delivery within 24 h	0.83 [0.72, 0.96]	22
Delivery within 48 h	0.89 [0.59; 1.35]	3
Interval to delivery	3.46 [1.85; 5.06]	32
Cesarean section rates	0.97 [0.86, 1.11]	42
Uterine tachysystole rates	0.73 [0.50; 1.04]	27
Postpartum hemorrhage	0.77 [0.45; 1.31]	8
Admission to NICU	1.09 [0.91; 1.30]	24
Umbilical cord pH < 7.2	0.65 [0.26; 1.61]	7
Meconium-stained AF	1.11 [0.92; 1.35]	25
5′ Apgar score < 7	0.81 [0.59; 1.11]	22
Sublingual misoprostol compared to vaginal		
Delivery within 24 h	1.10 [0.98; 1.24]	6
Delivery within 48 h	1.26 [0.37; 4.21]	2
Interval to delivery	– 1.11 [– 2.06; – 0.17]	13
Cesarean section rates	0.76 [0.56; 1.03]	14
Uterine tachysystole rates	0.67 [0.20; 2.20]	8
Postpartum hemorrhage	3.00 [0.12; 72.59]	1
Admission to NICU	1.00 [0.68; 1.47]	10
Umbilical cord pH < 7.2	1.26 [0.00; 375.47]	2
Meconium-stained AF	0.97 [0.75; 1.25]	8
5′ Apgar score < 7	0.92 [0.43; 1.94]	8

**Table 2:** Subgroup analysis of outcomes according to the maximum dose delivered

Outcome	RR/MD (95% CI)	Prediction intervals	Subgroup according to maximum dose		
			Low	Intermediate	High
Delivery within 24 h	0.90 (0.80, 1.01)	0.49, 1.63	–	–	–
Delivery within 48 h	1.02 (0.76, 1.38)	0.46, 2.25	–	–	–
Interval to delivery	1.95 (0.63, 3.27)	– 6.5, 10.46	1.09 (– 4.87, 7.06)	2.20 (0.64. 3.75)	1.83 (– 1.25, 4.92)
Cesarean section rates	0.92 (0.82, 1.04)	0.43, 1.99	0.88 (0.49, 1.60)	0.94 (0.81, 1.08)	0.89 (0.75, 1.07)
Uterine tachysystole rates	0.73 (0.52, 1.02)	0.12, 4.35	0.30 (0.14, 0.66)	0.74 (0.46, 1.18)	1.00 (0.54, 1.86)
Postpartum hemorrhage	0.77 (0.50, 1.18)	0.22, 2.64	1.00 (0.20, 4.85)	1.01 (0.28, 3.58)	0.73 (0.40, 1.32)
Admission to NICU	1.12 (0.91, 1.37)	0.38, 3.31	0.96 (0.51, 1.81)	1.20 (0.86, 1.68)	1.10 (0.83, 1.46)
Umbilical cord pH < 7.2	0.74 (0.42, 1.33)	0.12, 4.57	–	0.46 (0.15, 1.48)	1.18 (0.29, 4.72)
Meconium-stained AF	1.07 (0.92, 1.24)	0.49, 2.32	0.97 (0.58, 1.62)	1.13 (0.94, 1.35)	1.06 (0.91, 1.24)
5′ Apgar score < 7	0.79 (0.59, 1.06)	0.22, 2.91	0.74 (0.29, 1.89)	0.90 (0.59, 1.36)	0.66 (0.40, 1.12)

Subgroup analysis of secondary outcomes according to the maximum dose delivered in each study (Low dose = both groups received a dose of < 50 mcg of misoprostol, Intermediate = at least one of the two groups received = 50 < 100 mcg of misoprostol, High dose = at least one of the two groups received ≥ 100 mcg of misoprostol). (CI NA = Confidence intervals were not available following analysis). All secondary analyses (prediction intervals, subgroup) are based on the sum of studies

**Fig. 2:**
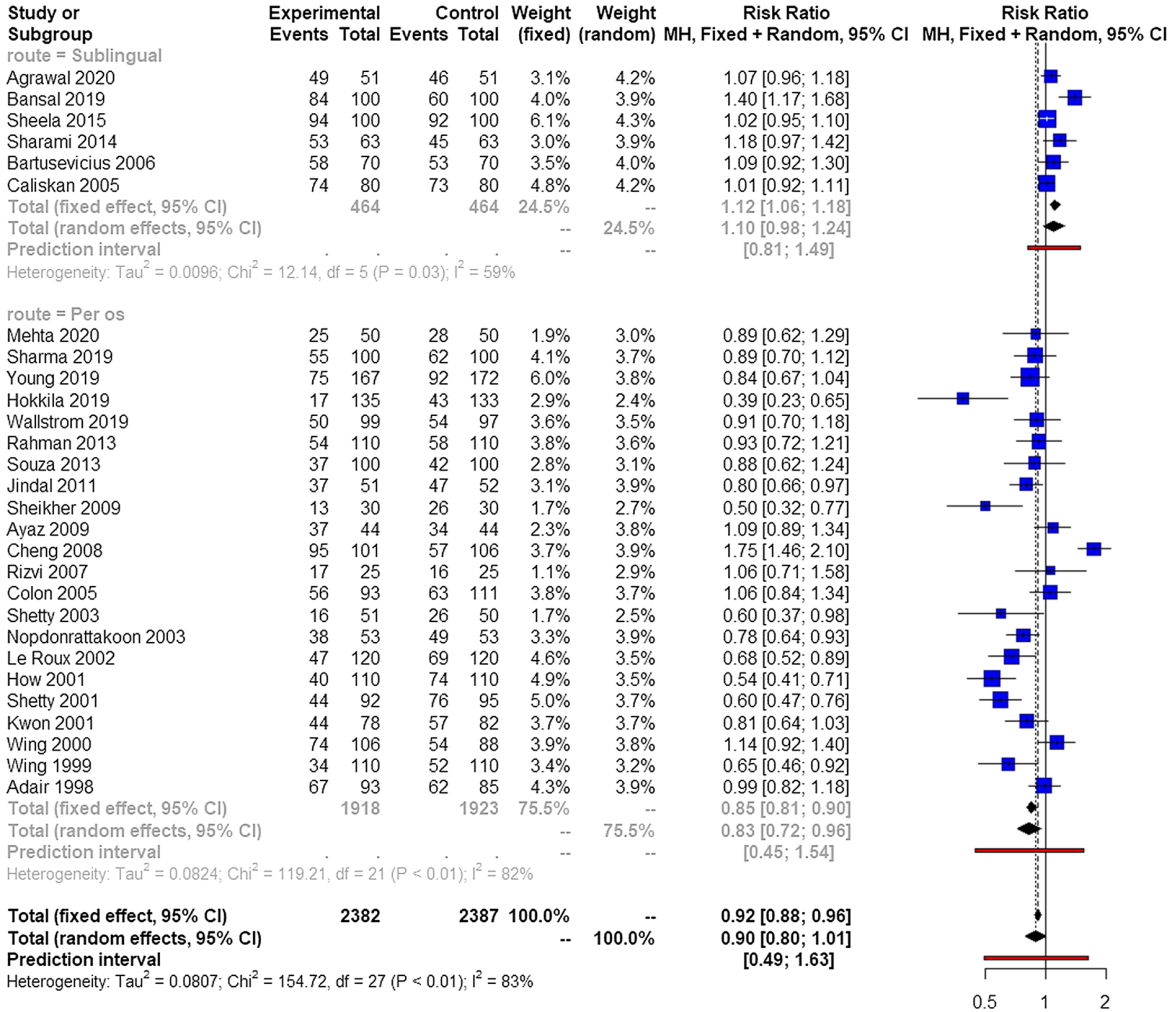
Forest plots of vaginal delivery within 24 h from onset of induction among parturient induced with oral misoprostol (subgrouped to sublingual and per os intake) and those induced with vaginally inserted misoprostol. (Vertical line = "no difference" point between the two groups. Blue squares = risk ratios; Diamonds = pooled risk ratios and 95 confidence intervals for all studies; Horizontal black lines = 95% CI; Horizontal red line = pooled 95% prediction intervals)

The various methods that were used were comparable in terms of successful vaginal delivery within 24 h (Fig. 2). Significant heterogeneity was observed among included studies. Small studies seem to influence the results and Rucker's meta-analysis revealed significant differences among the two groups in favor of the oral route (Table 3). Outlier analysis also indicated that several studies affected the overall effect with a *p* value closer to statistical significance for the adjusted estimate (*p* =  0.0857).

**Table 3:** Trim and fill analysis and p-curve analysis

Trim-fill analysis/outcome	MD/RR (95% CI)	Added studies	*p* value	Meta-regression, *p*-curve and small study effects analysis		
				Meta-regression	Evidential value	Rucker's analysis
Delivery within 24 h	1.05 (0.91, 1.20)	9	0.520	0.694	Present	1.13 (1.05, 1.22)
Delivery within 48 h	1.02 (0.76, 1.38)	0	0.007	0.435	–	1.32 (0.91, 1.91)
Interval to delivery	0.66 (-0.97, 2.29)	7	0.417	0.818	Present	0.99 (0.75, 1.23)
Cesarean section rates	1.02 (0.88, 1.17)	7	0.818	0.879	Present	1.00 (0.86, 1.16)
Postpartum hemorrhage	0.77 (0.50, 1.18)	0	0.670	0.683	–	0.72 (CI NA)
Admission to NICU	1.06 (0.86, 1.31)	3	0.589	0.986	–	1.18 (CI NA)
Uterine tachysystole rates	0.90 (0.62, 1.30)	6	0.568	0.455	–	0.80 (CI NA)
Umbilical cord pH < 7.2	1.04 (0.46, 2.36)	3	0.922	0.892	–	1.82 (0.35, 9.45)
Meconium-stained AF	1.06 (0.90, 1.24)	2	0.474	0.314	–	1.00 (0.75, 1.35)
5′ Apgar score < 7	0.79 (0.59, 1.06)	0	0.113	0.879	–	0.81 (CI NA)

Meta-regression analysis was based on the difference of the dosage between the two groups (Misoprostol dosage in the per os group minus the dosage that was delivered in the vaginal group). Trim and fill, *p*-curve analysis and Rucker's analysis are based on the sum of the studies that were included. *P*-curve analysis was omitted in outcomes that were not based in at least 2 studies that indicated a potentially significant (*p* < *0.05*) of the route of misoprostol intake. (CI NA = Confidence intervals were not available following analysis). All secondary analyses (trim-fill, meta-regression, *p*-curve and Rucker's analysis for small study effects) are based on the sum of studies

**Fig. 3:**
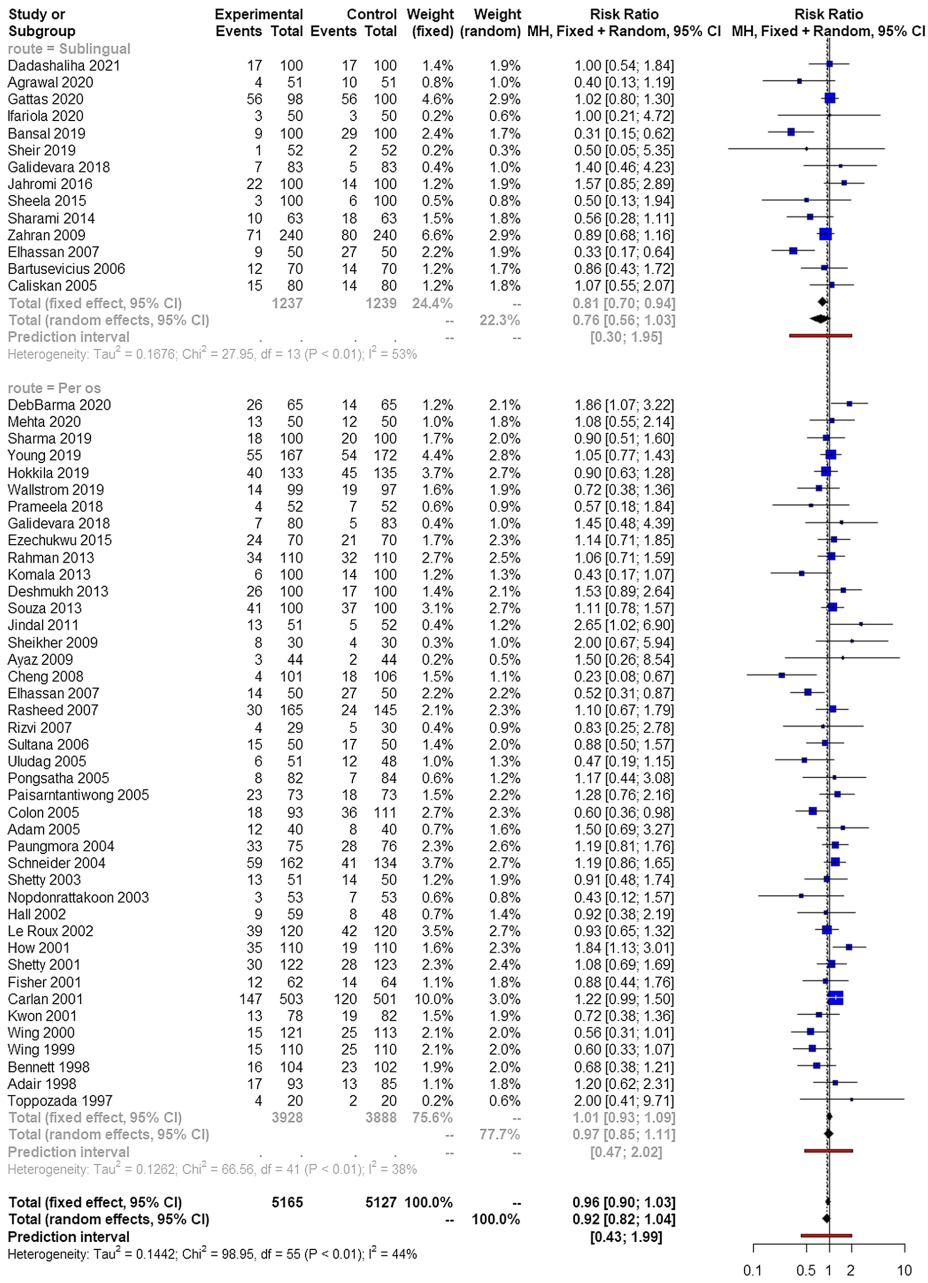
Forest plots of risk of delivering with cesarean section among parturient induced with oral misoprostol (subgrouped to sublingual and per os intake) and those induced with vaginally inserted misoprostol. (Vertical line = "no difference" point between the two groups. Blue squares = risk ratios; Diamonds = pooled risk ratios and 95 confidence intervals for all studies; Horizontal black lines = 95% CI; Horizontal red line = pooled 95% prediction intervals)

Cesarean section rates were comparable among the various routes of administration of misoprostol (Fig. 3). The dose of misoprostol did not influence these findings (RR 0.88, 95% CI 0.49, 1.60 for low doses, RR 0.94, 95% CI 0.81, 1.08 for intermediate doses and RR 0.89, 95% CI 0.75, 1.07 for high doses). Outliers, small study effects and meta-regression analysis did not influence the overall effect estimate.

Summary effect sizes of secondary outcomes are presented in Table 2. Briefly, the route of misoprostol intake did not influence delivery rates within 48 h from induction of labor or interval to delivery. Similarly, uterine tachysystole rates, risk of postpartum hemorrhage and risk of disseminated intravascular coagulation (DIC) were not affected by the route of administration. Concerning neonatal outcomes, risk of admission to the neonatal intensive care unit (NICU) was comparable among cases that received oral misoprostol to those that had intravaginal placement.

Of note, the interval to delivery was significantly reduced with sublingual misoprostol compared to vaginal (MD – 1.11 h, 95% CI – 2.06, – 0.17) and increased with per os misoprostol compared to vaginal (MD 3.45 h, 95% CI 1.85, 5.06) (Table 1).

### Publication bias

The significance of the results of secondary outcomes were not altered by the trim-fill and small study effects (Rucker's) analysis. Contour enhanced funnel plots did not reveal significant bias with the exception of the uterine tachysystole outcome (“Appendix”). Trim and fill analysis did not reveal significant alterations in the level of statistical significance of results (Table 3).

### Quality of evidence

The overall quality of the evidence for the evaluation of misoprostol's efficacy in successfully inducing labor and resulting in normal delivery was evaluated as high due to the low study limitations, very low possibility of indirectness and inconsistency of results, precision of findings and low risk of publication bias.

## Discussion

### Principal findings

The findings of our meta-analysis suggest that vaginal misoprostol increases the odds of successful vaginal delivery within 24 h compared to per os intake. However, sublingual intake might be superior to vaginal misoprostol. Of note, in terms of absolute time, sublingual misoprostol seems to be superior as it reduces the actual time from induction to delivery by approximately 1.11 h (compared to vaginal intake), whereas per os misoprostol seems to be the less effective mode as it increases this interval by approximately 3.45 h (compared to vaginal intake). However, direct comparison of these two modes of administration was not available in a substantial number of studies; hence, a network meta-analysis was not undertaken to evaluate the optimal route of intake.

### Comparison with existing literature

A previous meta-analysis that investigated differences in labour outcomes among women receiving sublingual and vaginal misoprostol revealed increased rates of uterine tachysystole in the sublingual group; however, its findings were based in 5 studies [8], whereas our analysis included outcomes from 39 studies (10 of them regarding sublingual intake) and revealed that both oral (either per os or sublingual) and vaginal intake are equally safe in inducing labour. In 2001 Crane et al. in a retrospective cohort of 519 women observed that the incidence of tachysystole and hyperstimulation varied among the various routes of misoprostol intake [71]. The results of the present meta-analysis; however, seem to contradict this and leave plenty of space for decision making in clinical practice.

In 2014 Alfirevic et al. conducted a meta-analysis and concluded that the appropriate dose of misoprostol intake should be considered the one that ranges between 20 and 25 mcg and that it is preferable to use oral intake compared to vaginal, especially in the setting of developing countries in order to help reduce the risk of maternal and neonatal infection. Our meta-analysis did not reveal substantial differences among the three groups in terms of safety, however, it should be noted that between subgroups (Low, intermediate, high doses), substantial heterogeneity was noted that revealed a trend towards increased intervals form induction to delivery in higher and intermediate doses, whereas lower doses had a comparable effect estimate compared to vaginal misoprostol. To date, there is limited evidence on the actual impact of the vaginal route on maternal and neonatal infection rates (including chorioamnionitis, post-partum endometritis, neonatal infection and neonatal sepsis) and given the indirectness of reporting meta-analysis was not performed for these outcomes.

### Strengths and limitations

The present meta-analysis is based in a large number of randomized controlled trials of low-moderate risk of bias. The secondary analyses that were performed permit safe interpretation concerning the accuracy of estimated effect sizes and estimation of risk of bias. Publication bias was thoroughly evaluated and indicated that our analysis is based mainly on studies of low-moderate risk of bias. Furthermore, for the first time it was found that the dose of misoprostol does not seem to affect rates of normal delivery (within 24- and 48-h).

On the other hand, several other confounders may partially limit the findings of this study, including co-administration of oxytocin for induction and augmentation of labor, parity, Bishop score at start of induction, presence of antenatal pathology (that may result in antepartum fetal distress, therefore, resulting in cesarean section) and gestational week at induction. However, meta-analyses that are based on aggregate data are always prone to these factors. In our analysis the Hartung-Knapp-Sidik-Jonkman random effects model was used which outperforms the traditional DerSimonian-Laird model as it consistently results in more adequate error rates when heterogeneous studies are considered for analysis; hence, confounders can be partially overlooked [13].

### Conclusions and implications

Considering the results of the present meta-analysis it seems reasonable to conclude that sublingual misoprostol seems to offer better results in terms of a shorter interval to delivery and successful vaginal delivery within 24 h compared to vaginal misoprostol which seems to perform better than the per os intake (both as a pill or oral solution). Maternal and neonatal morbidity is not affected by either mode of intake. Intermediate doses can be acceptable according to the outcomes of the present study and seem to be the most efficacious in terms of shortening the interval to delivery.

Clinicians should be aware of these findings and could consider using misoprostol orally or sublingually instead of vaginally. Furthermore, there is need for future research, especially randomized controlled trials comparing oral and sublingual routes of administration as there is limited data on this direct comparison.

## Data Availability

The data that support this systematic review and meta-analysis are available from the corresponding author, upon reasonable request.

## Appendix

### Search plot

(See Tables 4, 5)

**Table 4:** Study characteristics

Author Year	Inclusion criteria	Exclusion criteria	Indications for induction	Misoprostol dose	Women participated	Primary outcome investigated
Dadashaliha (2021) [20]	1. Gestational age ≥ 37 w 2. Bishop score ≤ 5 3. Single live fetus 4. Cephalic presentation 5. Fetal weight < 4000 g 6. Primigravida	1. Contraindications for the use of PG analogues 2. Abnormal fetal heart rate 3. Previous uterine scars 4. PROM, Placenta Previa, Placenta abruption 5. Fetal malformations 6. Multiparity 7. Signs of active labor at admission 8. Severe preeclampsia	1. Prolonged pregnancy	50 μg sublingually 50 mg vaginally	312 eligible 12 excluded 300 included	Evaluation of the safety and effectiveness of intracervical misoprostol in comparison with intravaginal and sublingual for the induction of labor at term
Mehta (2020) [27]	1. Gestational age ≥ 37 w 2. Bishop score < 6 3. Single live fetus 4. Cephalic presentation 5. Reactive non stress test 6. Clinically adequate pelvis	1. Contraindications for the use of PG analogues 2. Abnormal fetal heart rate 3. Previous uterine scars 4. Fetal malformations 5. Multiparity 6. Placenta previa 7. Bishop score > 6 8. Gestational age < 37w 9. Cephalo-pelvic disproportion 10. Multiple gestation	1. Prolonged pregnancy 2. PROM 3. Oligohydramnios 4. Preeclampsia 5. IUGR	25 µg orally 25 μg vaginally Maximum of five doses	100 included	Compare the effect of oral misoprostol 25 µg and vaginal misoprostol 25 µg for induction of labor at term
Gattás 2018 [22]	1. Gestational age ≥ 37 w 2. Bishop score ≤ 6 3. Single live fetus 4. Cephalic presentation 5. Estimated fetal weight < 4000 g 6. Amniotic fluid index > 5	1. Previous uterine scar 2. Chorioamnionitis 3. Genital bleeding 4. Fetal abnormalities 5. Tumors 6. Contraindications to vaginal delivery 7. HIV positivity	1. Prolonged pregnancy 2. PROM 3. Gestational hypertension 4. Gestational diabetes	12.5 μg sublingually 25 μg vaginally	450 eligible 250 excluded 2 refused to continue 198 included	Compare the frequency of tachysystole when misoprostol is administered sublingually at the dose of 12.5 μg versus vaginally at a dose of 25 μg to induce labor in a full-term pregnancy with a live fetus
Ifariola (2020) [24]	1. Gestational age ≥ 37 w 2. Bishop score ≤ 5 3. Single live fetus 4. Cephalic presentation 5. Normal fetal heart rate	1. Contraindications for the use of PG analogues 2. Abnormal fetal heart rate 3. Previous uterine scar 4. Chorioamnionitis 5. Malpresentation 6. Vaginal bleeding 7. Placenta praevia 8. Estimated fetal weight of > 4000 g 9. Cephalopelvic disproportion 10. Pre-existing medical illness 11. Grand multiparty 12. Nonconsenting patients 13. Preeclampsia/eclampsia	1. Prolonged pregnancy 2. PROM 3. Gestational hypertension 4. Suspected intrauterine growth restriction 5. Chronic hypertension and diabetes	25 µg sublingually 25 µg vaginally	100 who fulfill the inclusion criteria	Compare the efficacy of vaginally administered with sublingual misoprostol for cervical ripening and labor induction
Agrawal (2020) [15]	1. Gestational age ≥ 37 w 2. Single live fetus 3. Cephalic presentation 4. Estimated fetal weight < 4000 g 5. Intact membrane	1. Previous uterine scars 2. Cephalopelvic disproportion 3. Twin pregnancy 4. Malpresentation 5. Pre-existing medical illness 6. AFI < 5 7. P.R.O.M 8. Antepartum haemorrhage	NA	25 µg sublingually 25 µg vaginally	102 included	Compare the efficacy of sublingual versus vaginal misoprostol for induction of labour in primigravida at term
DebBarma (2020) [70]	1.Single live fetus 2.Cephalic presentation 3.Gestaional age > 37w 4.Bishop score < 5 5. Intact membrane 6. Without previous stripping of membranes	1. Cephalopelvic disproportion 2. Previous uterine scar 3. Multiple gestations 4. Malpresentation 5. Contraindications for the use of PG analogues 6. Contraindications for vaginal delivery 7. Chorioamnionitis 8. Suspected fetal jeopardy 9. Placenta previa 10. Drug allergy 11. Grand multigravida	1. Prolonged pregnancy 2. Preeclampsia 3. Prolonged pregnancy with preeclampsia 4. Olygohydramnios 5. Elective 6. IHCP	25 μg orally 25 μg vaginally Every 4 h Maximum of 5 doses	130 included	Compare the time interval from induction to vaginal delivery between orally and vaginally administered misoprostol
Wallstrom (2019) [33]	1. Gestational age ≥ 37 w 2. Bishop score ≤ 4 3. Single live fetus 4. Cephalic presentation 5. Primigravida	1. Abnormal fetal heart rate 2. Previous uterine scars 3. Language barriers 4. Intrauterine growth restriction (IUGR)	1. Prolonged pregnancy 2. PROM	25 µg orally 7 µg/hour vaginally	912 eligible 196 included	Compare the WHO recommended orally administrated dosage of misoprostol (25 μg) with a vaginal slow-release (7 μg/hour) insert of misoprostol regarding time from induction to delivery and safety of the method
Bansal (2019) [16]	1. Gestational age ≥ 37 w 2. Bishop score < 6 3. Single live fetus 4. Cephalic presentation 5. Intact or early rupture of membranes 6. Reactive foetal heart pattern	1. Previous uterine scar 2. Malpresentations 3. Cephalopelvic disproportion 4. Placenta praevia, vasa praevia, Contracted pelvis 5. Pre-existing medical illness	1. Prolonged pregnancy 2. PROM 3. Gestational hypertension 4. Oligohydramnios 5. Foetal growth restriction 6. Maternal medical illness requiring termination 7. chronic hypertension 8. Intrauterine foetal death	25 µg sublingually 25 µg vaginally	200 included	Compare the previously used vaginal administration of Misoprostol with sublingual administration for labor induction regarding induction delivery interval, maternal and neonatal outcome
Sharma (2019) [44]	1. SIngleton pregnancy 2. Gestational age > 37w 3. Clinically adequate pelvis 4. Bishop score < 6 5. Reactive NST 6. Absence of uterine contractions	1. Malpresentation 2. Presence of uterine contractions ≥ 3/10 min 3. Cephalopelvic disproportion 4. Bishop score > 6 5. Previous uterine scar 6. Multiple gestation 7. Placenta previa 8. Non reactive NST 9. Contraindication for vaginal delivery 10. Hypersensitivity to PG 11. Parity -5 or more	1. Prolonged pregnancy 2. IUGR 3. Severe preeclampsia 4. Oligohydramnios 5. Intrauterine fetal death 6. Eclampsia 7. Congenital anomalies	50 mg orally every 4 h for maximum 5 doses 25 μg vaginally every 4 h for maximum 5 doses	200 included	Compare mode of delivery, induction to delivery interval in vaginal delivery and vaginal delivery when with vaginally used vs orally used misoprostol
Sheir (2019) [32]	1. Gestational age ≥ 37w 2. Bishop score ≤ 5 3. Single live fetus 4. P.R.O.M 5. Reactive cardiotocographics trace 6. Primigravida 7. Oligohydramnios (AFI < 5) 8. Clinically adequate pelvis	1.Contraindications for the use of PG analogues 2. Abnormal fetal heart rate 3. Previous uterine scar 4. Malpresentation 5. Grand mutlipara 6. Pre-existing medical illness 7. Maternal or fetal factors 8. Contraindications for induction of labor	NA	50 μg sublingually 50 μg vaginally	104 included	Compare 50 μg of sublingual misoprostol to 50 μg of vaginal misoprostol for induction of labour at term regarding efficacy and safety
Hokkila (2019) [23]	1. Bishop score < 6 2. Single live fetus 3. Cephalic presentation 4. Nulliparous women at term	1. Previous uterine scar 2. Intrauterine growth restriction 3. Gestational age ≤ 36 4. PROM, placenta previa 5. Language barriers 6. Severe preeclampsia	1. Prolonged pregnancy 2. Gestational hypertension 3. Gestational diabetes 4. Oligohydramnion 5. Fetal macrosomia 6.Maternal exhaustion 7. Cholestasis of pregnancy	50 μg orally increased to 100 μg if needed 200 μg vaginally	283 included	Compare the efficacy of a 200‐μg misoprostol vaginal insert vs oral misoprostol regarding the cesarean section rate and the time interval to vaginal delivery in nulliparous women with unfavorable cervix
Young (2020) [35]	1. Gestational age > 37w 2. Single live fetus 3. Cephalic presentation	1.Contraindications for the use of PG analogues 2. Abnormal fetal heart rate 3. Previous uterine scar 4. Uncontrolled epilepsy or asthma 5. Contraindication to vaginal birth	1. Prolonged pregnancy 2. PROM 3. Maternal hypertension	50 μg orally every 4 h 25 to 50 μg vaginally every 6 h	586 eligible 511 included	Compare the effectiveness and safety of oral misoprostol, low dose vaginal misoprostol and our established dinoprostone vaginal gel for induction
Khan (2018) [26]	1. Gestational age ≥ 37w 2. Single live fetus 3. Estimated fetal weight > 2500 g	1. Contraindications for the use of PG analogues 2. Abnormal fetal heart rate 3. Previous uterine scar 4. Congenital fetal abnormality 5. Fetal growth restriction 6. Oligohydramnios	1. Prolonged pregnancy	Maximum dose of 150 mcg (6 doses)	272 included	Compare the efficacy of misoprostol, oral and vaginal when administered for induction of labor at term live pregnancy
Galidevara (2018) [21]	1. Gestational age > 34 w 2. Bishop score ≤ 6 3. Single live fetus 4. Cephalic presentation 5. PROM 6. Reassuring non-stress test	1. Contraindications for the use of PG analogues 2. Previous uterine scar 3. Chorioamnionitis 4. Cephalopelvic disproportion 5. Major fetal anomalies 6. Women in established labour 7. Suspected macrosomia 8. Antepartum hemorrhage 9. Active genital infection	1. PROM after 34 weeks of gestation	50 µg orally 25 μg vaginally 50 μg sublingually	246 included	Evaluation of the efficacy and safety of different routes of administration of misoprostol for induction of labour in women with premature rupture of membranes after 34 weeks of gestation
Prameela (2018) [57]	1. Single live fetus 2. Gestational age 37–42 w 3. Nulliparous women 4. Cephalic presentation 5. Postdated pregnancy 6. PROM 7. Bishop score < 6	1. Previous uterine scar 2. Cephalopelvic disproportion 3. Antepartum haemorrhage 4. Multiparity 5. Multiple gestation 6. Oligohydramnios 7. Polyhydramnios 8. IUGR 9. Medical disorders (eg DM, hypertension) 10. Contraindication to PG 11. PPROM 12. History of glaucoma and epilepsy	1. Prolonged pregnancy 2. PROM	100 μg orally 25 μg vaginally every 4 h, maximum of 6 doses	104 included	Comparison between use of oral vs vaginal misoprostol for induction of labour at term and neonatal outcome
Madhu (2017)	1. Singleton pregnancy 2. Gestational age > 37w 3. Cephalic presentation 4. Bishop score ≤ 5 5. Good fetal cardiac activity	1. Previous uterine scar 2. Malpresentation 3. ≥ G3 3.Fetal distress 4. Contraindication for PG use 5. Placenta previa 6. Cephalopelvic disproportion	1. Postdated pregnancy 2. PROM 3.Preeclampsia/hypertension 4. Eclampsia 5. IUGR	25 μg sublingually 25 μg vaginally Every 4 h, maximum of 6 doses	100 included	Efficacy of sublingual vs vaginal misoprostol administration in terms of successful vaginal delivery, induction delivery time interval, number of doses required, need for oxytocin augmentation and fetal outcome measures
Jahromi (2016) [25]	1. Gestational age ≥ 37w 2. Bishop score < 7 3. Single live fetus 4. Cephalic presentation 5. Estimated fetal weight < 4000 g	1. Contraindications for the use of PG analogues 2. Abnormal fetal heart rate 3. Previous uterine scar 4. Malpresentation 5. Fetal congenital malformations 6. Intrauterine growth restriction 7. Gestational age < 37w 8. Bishop score = 8 9. Oligohydramnios, placenta previa	3rd trimester of pregnancy with any obstetric or medical indications for the induction of labor	25 μg sublingually 25 μg vaginally Every 4 h until the cervical Bishop score was > 8 Maximum dose of 150 μg of misoprostol	474 assessed for eligibility 274 excluded 200 included	Compare the effectiveness and safety of sublingual versus vaginal misoprostol for the termination of pregnancy with a live full-term fetus
Ezechukwu (2015) [56]	1. Uncomplicated singleton pregnancy 2. cephalic presentation 3. Gestational age 37–42 w 4. Absence of uterine contractions	1. Unsure of date 2. Pre-conception irregular menstrual cycle 3. Contraindication to vaginal delivery 4. Medical diseases in pregnancy 5. PROM 6. Contraindication to PG use (allergy, asthma, glaucoma, previous CS)	N/A	50 μg vaginally 50 μg orally Every 6 h, maximum of 4 doses	153eligible 140 included (10 exclusian criteria, 3 refusal to give consent)	Compare induction-vaginal delivery inteval, route of deliver, Apgar score at birth, need for oxytocine augmentation, maternal satisfaction with the route of administration of vaginal vs oral misoprostol for induction of labor
Sheela (2015) [31]	1. Single live fetus 2. Cephalic presentation 3. Reassuring foetal heart rate (FHR) pattern	1. Contraindications for the use of PG analogues 2. Previous uterine scar 3. Multiparity	1. Singleton term pregnancy	50 μg sublingually 25 μg vaginally	200 included	Compare the safety and efficacy of 50 μg of sublingual Misoprostol with those of 25 μg of vaginal Misoprostol for induction of labour at term
Sharami (2014) [30]	1. Gestational age ≥ 36 2. Bishop score ≤ 4 3. Single live fetus 4. Cephalic presentation 5. Estimated fetal weight < 4000 g 6. Normal fetal heart rate 7. Intact membranes 8. Nulliparous	1. Contraindications for the use of PG analogues 2. Previous uterine scar 3. Cephalopelvic disproportion 4. Preeclampsia 5. Fetal heart rate abnormalities 6. Contraindications to vaginal delivery	1. Gestation > 41 weeks 2. Oligohydramnios 3. Gestational diabetes 4. Intrauterine growth restriction	25 μg sublingually 50 μg vaginally	131 eligible 126 included	Compare the effectiveness and safety of 25 μg sublingual misoprostol dose vs. standard dose (50 μg) of intravaginal misoprostol for cervical ripening and labor induction
Deshmukh (2013) [45]	1. Primigravida 2. Gestational age 34–42 w 3. Singleton viable pregnancy 4. Bishop score = < 5 5. Cephalic presentation 6. Clinically adequate pelvis 7. Reactive NST	1. Hypersensitivity or any contraindication to the use of PG 2. Antenatal complication necesssitating CS 3. Patients refusal to give consent	N/A	50 μg vaginally 50 μg orally Every 6 h for maximum 4 doses	200 included	Compare the efficacy of oral with vaginal misoprostol for induction of labour
Komala (2013) [38]	1. Singleton pregnancy 2. Cephalic presentation 3. Gravida 1 to 4 4. Clinically adequate pelvis 5. Bishop score < 6 6. Pregnancy induced hypertension 7. Pre-eclampsia 8. Antepartum eclampsia 9. Prolonged pregnancy 10. Olygohydramnios 11. PROM	1. Severe systematic ilnesses (uncontrolled DM, preeclampsia, cardiac, renalorhepatic disease) 2. Intrauterine deaths 3. Hypersensitivity to misoprostol or PG analogue 4. Contraindication to induction and vaginal delivery (cephalopelvic disproportion, malpresentation, foetal compromise, no reassuring foetal heart rate pattern, previous scar, antepartum haemorrhage)	1. Prolonged pregnancy 2. Pregnancy induced hypertension 3. PROM 4. Olygohydramnios 5. Preeclampsia 6. Antepartum eclampsia	25 μg vaginally every 4 h, maximum of 4 doses 2tbs of 25 μg orally every 4 h, maximum of 4 doses	200 included	Time interval from induction to vaginal deliveryand vaginal delivery rate within 24 h
Souza (2013) [37]	1. Gestational age ≥ 37w 2. Bishop score ≤ 6 3. Single live fetus 4. Cephalic presentation 5. Estimated fetal weight < 4000 g 6. Amniotic fluid index > 5 cm	1. Abnormal fetal heart rate 2. Previous uterine scar 3. Fetal abnormalities 4. Restricted fetal growth 5. Genital bleeding related to placenta 6. Contraindications to vaginal delivery	1. Gestation > 41 weeks 2. PROM 3. Gestational hypertension 4. Gestational diabetes	Vaginal misoprostol tablets 25 μg of misoprostol every 6 h for a maximum of 8 doses Oral misoprostol solution Initial misoprostol dose 20 μg/hour; dose increased by 20 μg/hour every 6 h up to 80 μg/hour for a maximum of 48 doses	200 included	Determination of the efficacy and safety of a titrated oral misoprostol solution compared with vaginal misoprostol tablets for labor induction
Rahman (2013) [48]	1. Multiparous or nulliparous 2. Singleton pregnancy 3. Gestational age 37-42w 4. Bishop score ≤ 6 5. Cephalic presentation 6. Reactive FHR pattern 7. Absence of contractions	1. Malpresentation 2. Uterine contractions 3. Cephalopelvic disproportion 4. EFW > 4000gr 5. Maternal age < 18yo 6. Parity ≥ 5 7. Bishop scpre > 6 8. Previous CS or uterine scar 9. Multiple gestation 10. Undiagosed vaginal bleeding 11. Fetal distress 12. Suspected chorioamnionitis 13. Contraindications to vaginal delivery or administration of PG	1. Postdates 2. PROM 3. Pregnancy induced hypertension (PIH) 4. Postdates and PIH 5. OTher	50 μg orally 25 μg vaginally Every 4 h, maximum of 5 doses	228 randomised 220 included	Assess and compare the efficacy and safety of 50 μg oral misoprostol ad 25 μg intravaginal misoprostol for induction of labour at term
Jindal (2011) [52]	1. Term gestation 2. Singleton pregnancy 3. Cephalic presentation 4. No fetal congenital malformation 5. Reactive FHR pattern 6. Bishop score ≤ 4 7. Rupture of membranes < 4 h duration	1. Bishop scpre > 4 2. Cephalopelvic disproportion 3. Placenta previa 4. Unexplained vaginal bleeding 5. Previous CS or uterine scar 6. Active herpes simplex 7. Carcinoma cervix 8. Chorioamnionitis 9. Contraindication to use of PG	1. Hypertensive disorders 2. IUGR 3. Olygohydramnios Diabetes mellitus	50 μg vaginally 50μh orally Every 4 h, maximum of 6 doses	110 recruited 103 included	Compare efficacy and safety of 50 μg misoprostol vaginal with oral for labor induction
Mehrotra (2010) [47]	1. Singleton pregnancy 2. Gestational age ≥ 37 3. Bishop score ≤ 6 4. Intact membranes 5. Cephalic presentation 6. Reactive NST	1. Contraindication to vaginal birth 2. Previous uterine surgery 3. Abnormal FHR pattern 4. Ruptured membranes 5. Vaginal bleeding 6. Hypersensitivity to misoprostol or PG analogues	N/A	50 μg vaginally 50 μg orally Every 4 h maximum up to 200 μg	128 included (68 vaginal misoprostol, 60 oral misoprostol)	Time of intervention to vaginal birth and dose of misoprostol required in each group
Ayaz (2009) [39]	1. Age 26–40 2. Multigravida 3. Accurate dating of gestation 4. Gestational age 40–42 w 5. Cephalic presentation 6. Bishop score < 6 7. Intact membranes 8. Patients height > 150 cm	1. Contraindications to use of PG 2. Placenta previa 3. Previous uterine surgery 4. Antenatal complications	N/A	50 μg vaginally 50 μg orally Every 4 h, maximum of 6 doses	88 included	Time from induction to onset of significant uterine contractions and induction to delivery
Zahran (2009) [36]	1. Gestational age ≥ 37w 2. Bishop score ≤ 4 3. Single live fetus 4. Cephalic presentation 5. Reactive non-stress test 6. Amniotic fluid index ≥ 5 cm	1. Previous uterine scar 2. Fetal abnormalities 3. Fetal weight > 4000 g 4. Intrauterine growth restriction 5. PROM 6. Cephalopelvic disproportion 7. Contraindication to vaginal delivery	1. Prolonged pregnancy 2. Gestational hypertension 3. Antepartum fetal distress	50 μg sublingually 50 μg vaginally Every 6 h for 24 h	480 included	To assess the effectiveness and safety of sublingual misoprostol (50 mg), compared with the same dose administered vaginally every 6 h for cervical ripening and labor induction in women with a viable fetus in the third trimester of pregnancy
Sheikher (2009) [69]	1. Gestational age 37–42 w 2. Cephalic presentation 3. Primi or second gravidae 4. Bishop score < 7 5. Absence of uterine contractions	1. Previous uterine incision 2. Non reassuring FHR 3. IUGR 4. Oligohydramnios 5. Placenta previa 6. Evidence of chorioamnionitis 7. Active herpes infection 8. EFW > 4 kg 9. Renal or hepatic disease	1. Prolonged pregnancy 2. Hypertensive disorders 3. Congenital anomalies	50 μg orally 50 μg vaginally	60 inluded	N/A
Cheng (2008) [63]	1. Gestational age 34–42 w 2. Singleton pregnancy 3. Bishop score ≤ 6 4. Cephalic presentation 5. Reassuring FHR pattern	1. Non reassuring FHR pattern 2. Parity > 5 3. Contraindication to labor and/or vaginal delivery 4. Uterine scar 5. Suspected plaacental abruption with abnormal FHR pattern 6. Vaginal bleeding 7. Cervical dilation > 4 cm 8. Uterine contractions ≥ 3/10 min 9. Significant maternal renal, cardiac or hepatic disease 10. Hypersensitivity to misoprostol or PG analogues	1. Prolonged pregnancy 2. Hypertension 3. IUGR 4. PROM 5. GDM 6. Oligohydramnios 7. Psychosocial	25 μg vaginally, every 4 h 20 mL oral solution (iμg/mL) every 1 h for 4 doses, increase to 40 μg every 1 h for 4 doses, increase to 60 μg every 1 h	207 included	Interval from first misoprostol dose to vaginal delivery and the percentage of women who delivered infants vaginally within 12 h vand 24 h of induction Incidence of tachysystole, hypertonus, uterine hyperstimulation, no reassuring FHR
Elhassan (2007) [46]	1. Single live fetus 2. Bishop score < 5	1. Uterine scar 2. Asthma 3. Heart disease 4. Parity > 6	1. Prolonged pregnancy 2. Preeclampsia 3. Hypertension 4. Diabetes mellitus 5. Antepartum hemorrhage 6. Others	50 μg vaginally 50 μg orally 50 μg sublingually	150 included	Induction to delivery time, CS rate, Apgar score at 1 min, presence of meconium-stained liquor, referral to pediatrician
Rasheed (2007) [29]	1. Gestational age ≥ 37w 2. Single live fetus 3. Cephalic presentation 4. Nulliparous and multiparous < para 4	1. Abnormal fetal heart rate 2. Previous uterine scar 3. Moderate to severe abruption 4. Placenta praevia	1. Prolonged pregnancy 2. PROM 3. IUGR 4. Oligohydramnios 5. Gestational hypertension 6. Prolonged latent phase 7. Mild antepartum haemorrhage (APH) without placenta praevia 8. Non reactive CTG 9. Bad obstetric history 10. Social reasons 11. Previous still birth at term 12. Precious pregnancy 13. Large baby with adequate maternal pelvis 14. Diminished foetal movement 15. Suspected congenital heart block 16. Sickle cell anemia	50 μg orally 50 μg vaginally Every 4–6 h to a maximum of 6 doses	310 included	Compare the safety and efficacy of oral versus vaginal misoprostol for induction of labour
Rizvi (2007) [67]	1. Primigravidae and second gravidae 2. Bishop score < 4	1. Ruptured membranes 2. Bishop score > 4 3. Previous uterine scar	1. Oligohydramnios 2. Preeclampsia 3. PROM 4. Diabetes mellitus 5. Postdate 6. Abnormal FHR 7. FGR	100 μg orally 25 μg vaginally Every 4 h to a maximum of 6 doses	60 included one withdrawn	Successful induction (vaginal delivery within 24 h of induction)
Bartusevicius (2006) [17]	1. Gestational age ≥ 41 w 2. Bishop score ≤ 6 3. Single live fetus 4. Cephalic presentation 5. Normal fetal heart rate	1. Contraindications for the use of PG analogues 2. Previous uterine scar 3. Chorioamnionitis 4. Ominous FHR, active uterine bleeding 5. Oligohydramnios 6. Fetal malformation 7. Macrosomia, Growth restriction 8. Multiparity	1. Prolonged pregnancy 2. Gestational hypertension 3. Gestational diabetes 4. PROM	50 μg sublingually 25 μg vaginally	418 eligible 280 excluded 138 included	Compare the efficacy and safety of 50 mg of sublingual misoprostol with 25 mg of vaginal misoprostol administered for labor induction at term
Sultana (2006) [55]	1. Gestational age 37–42 w 2. Gravida 1–3 3. Parity 0–2 4. Single live fetus 5. Cephalic presentation 6. Bishop score ≤ 4	N/A	1. Postdates 2. Preeclampsia/ eclampsia 3. Oligohydramnios 4. IUGR 5. Pregnancy induced hypertension 6. Others	100 μg vaginally 100 μg orally Every 4 h, maximum of 6 doses	100 included	Compare the efficacy and safety of oral and vaginal administration of misoprostol tablets for cervical ripening and induction of labour
Adam (2005) [49]	1. Siingleton pregnancy 2. Bishop score < 5	1. Uterine surgery 2. Antepartum hemorrhage 3. History of asthma, heart disease 4. Grand multiparity (≥ 7)	N/A	50 μg vaginally 50 μg orally Every 6 h, maximum of 4 doses	100 included	Induction to delivery interval, necessity of oxytocin augmentation, presence of meconium-stained liquor, cesarean delivery rate, Apgar score, referral to pediatrician
Colon (2005) [50]	1. Gestational age 32–42 w 2. Bishop score ≤ 6 3. Intact or ruptured membranes 4. Cephalic presentation Reassuring FHR pattern	1. Nonreassuring FHR pattern 2. Contraindication to labor or vaginal delivery 3. Uterine scar 4. Suspected placenta abruption with abnormal FHR pattern 5. Vaginal bleeding 6. Cervical dilation ≥ 4 7. Uterine contractions ≥ 3/10 min 8. Significant maternal cardiac/renal/hepatic disease 9. Maternal glaucoma 10. Hypersensitivity to misoprostol or PG	1. Postdares 2. Hypertension 3. Diabetes 4. Olygohydramnios 5. Others	25 μg vaginally every 4 h up to 4 doses 50 μg orally, followed by 100 μg every 4 h up to 4 doses	204 included	Interval from first misoprostol dose to delivery
Caliskan (2005) [19]	1. Bishop score < 5 2. Single live fetus 3. Cephalic presentation 4. Estimated fetal weight < 4500 g 5. Absence of spontaneous uterine contractions 6. Reactive nonstress test	1. Contraindications for the use of PG analogues 2. Previous uterine scar 3. Non-cephalic presentation 4. BMI ≥ 30 before conception 5. Previous attempt of labor induction for the present pregnancy	1. Gestational age > 41w 2. Intrauterine growth restriction 3. Oligohydramnios 4. Preeclampsia	50 μg sublingually 50 μg vaginally	160 included	Compare the efficacy of misoprostol 50 μg vaginally and 50 μg sublingually for labor induction at term
Paisarntantiwong (2005) [59]	1. Singleton pregnancy 2. Cephalic presentation 3. Reassuring FHR pattern 4. Bishop sore ≤ 6 5. Gestatioal age ≥ 41w without labor pain or ≥ 37w with indications for labor induction (eg hypertension, olygohydramnios, IUGR, gestational DM) 6. Intact membranes 7. Absence of uterine contraction (in a 20 min interval)	1. EFW > 4000gr or evidence of cephalopelvic disproportion 2. Parity > – 6 3. Previous CS or uterine incision 4. Contraindication to vaginal delivery (placenta previa, vasa previa, active genital herpes simples) 5. Evidence of chorioamnionitis (maternal temperature ≥ 100.4F, uterine tenderness and/or foul smelling amniotic fluid) 6. Contraindication for PG use (glaucoma or preexisting cardiac disease)	1. Gestational age > 42w 2. Gestational age > 41-42w 3. Olygohydramnios 4. Preeclampsia 5. Chronic hypertension 6. Gestational DM	25 μg vaginally 50 μg orally	146 included	Time interval from misoprostol administration to delivery nd the number of women required oxytocin augmentation for labor
Pongsatha (2005) [61]	1. Singleton pregnancy 2. Cephalic presentation 3. Gestational age 34–42 w 4. Bishop score ≤ 4 5. Intact membranes 6. Absence of labor or fetal distress 7. No previous uterine surgery 8. No obvious cephalopelvic disproportion 9. No contraindication for PG use	N/A	N/A	50 μg vaginally every 4 h 100 μg orally every 3 h Maximum dosing at 24 h	166 included	Time interal from induction to vaginal delivery
Uludag (2005) [62]	1. Singleton pregnancy 2. Cephalic presentation 3. Reactive NST 4. Bishop score < 6 5. Cervical dilation < 3 cm 6. Uterine contractions < 6/h	1. Uterine surgery 2. Contraindication to vaginal delivery 3. Parity > 5 4. Hyperxensitivity to PG	1. Postdates 2. Olygohydramnios 3. PROM 4. Preeclampsia 5. IUGR + olygohydramnios 6. Gestational diabetes 7. Other	50 μg vaginally 100 μg orally Every 4 h, maximum of 6 doses	99 included	Induction to vaginal delivery interval
Paungmora (2004) [28]	1. Gestational age ≥ 37w 2. Bishop score < 6 3. Single live fetus 4. Cephalic presentation 5. Intact membranes	1. Contraindications for the use of PG analogues 2. Previous uterine scar 3. Contraindications to vaginal birth	1. Prolonged pregnancy 2. Intrauterine growth restriction 3. Gestational diabetes 4. Gestational hypertension 5. SLE 6. Hypothyroid with fetal tachycardia 7. IgA nephropathy	100 μg orally 50 μg vaginally every 6 h for 48 h	153 included	Compare the efficacy of oral with vaginal misoprostol for induction of labor at term
Schneider (2004) (Congress abstract) [68]	N/A	N/A	N/A	50 μg orally 25 μg vaginally Every 4 h	311 included	N/A
Nopdonrattakoon (2003) [53]	1. Singleton pregancy 2. Parity ≤ 4 3. Cephalic presentation 4. Normal FHR tracing 5. Bishop score ≤ 4 6. No premature contraction 7. No PROm	1. Previous uterine surgery or CS 2. Significan fetal or medical concern 3. Contraindications to the use of PG	N/A	50 μg vaginally 50 μg orally Every 4 h, maximum of 6 doses	106 included	Induction to regular contraction interval and induction to vagianl delivery interval
Shetty (2003) [41]	1. Singleton pregnancy 2. Cephalic presentation 3. Gestational age ≥ 37w 4. Bishop score = < 8 5. Intact membranes	1. Previous CS 2. Hypersensitivity to PG 3. Parity > 5	1. Postdates 2. Hypertension/preeclampsia 3. Diabetes 4. Small baby 5. Others	25 μg vaginally 100 μg orally Every 4 h, maximum of 5 doses	101 included	Number of women delivering vaginally within 24 h of induction
Hall (2002) [58]	1. Singleton pregnancy 2. Gestational age > 37w 3. Cephalic presentation 4. No contraindication to vaginal delivery	1. Previous uterine surgery 2. PG hypersensitivity 3. Contractions > + 3/10 min 4. Nonreassurng FHR tracing 5. Contraindication to vaginal delivery	1. Postdates 2. Rupture of membranes 3. Preeclampsia 4. Oligohydramnios 5. Non reactive FHR tracing 6. IUGR 7. Diabetes 8. Other	25 μg vaginally 100 μg orally Every 3–4 h Repeated doses vaginal (25-50 μg) or orally (100-200 μg) util labor was established	107 included	Time from induction initiation to vaginal delivery
Le Roux (2002) [66]	1. Singleton pregnancy 2. Gestational age > 34w 3. Cephalic presentation 4. Unruptured membranes 5. Normal FHR tracing 6. Parturient age > 18	1. Previous CS 2. Parity > 4 3. Fetal anomaly 4. Fetal death 5. Bishop score > 7	NA	50 μg vaginally 50 μg orally Every 6 h to a maximum of 4 doses	573 recruited 93 withdrawn	Number of women delivering vaginally within 24 h of induction
Carlan (2001) [60]	1. Single live pregnancy 2. Bishop score < 7 3. EFW < 4500gr 4. Gestational age > 24w	1. Vaginal bleeding 2. Nonreassuring FHR 3. Breech presentation 4. Uterine contractions > 4/20 min 5. Labor 6. Contraindication to vaginal delivery	1. Hypertension 2. Postdates 3. Preeclampsia 4. Diabetes 5. Low amniotic fluid 6. Other or combination of reasons	50 μg vaginally for 2 doses, and increase to 100 μg, for maximum 500 μg total 200 μg orally for 2 doses and increase to 300 μg, for maximum 1600 μg total Doses given every 6 h	1004 included	Incidence of intervention
Fisher (2001) [64]	1. Bishop score < 9 2. Singleton pregnancy 3. Normal fetal anatomy with reactive NST 4. Cephalic presentation	1. Oligohydramnios (AFI < 2.5thpercentile for GA) 2. IUGR (EFW < 10^th^ percentile for GA) 3. Abruptio placenta 4. Prom 5. Previous uterine scar 6. Prior PG use for cervical ripening/labor induction 7. Contraindication to vaginal delivery	1. Postdates 2. Hypertension 3. Diabetes 4. Other	50 μg vaginally every 6 h 50 μg orally every 3 h Maximum of 48 h	126 included	Time from first misoprostol administration to delivery
Kwon (2001) [51]	1. Gestationa age 37–42 w	1. Previous uterine scar 2. Oligohydramnios 3. IUGR 4. PROM 5. Multifetal pregnancy 6. Cervix favourable for amniotomy (> 2 cm dilation with > 50% effacement, soft, mi-to-anterior position)	1. Postdates 2. Hypertension 3. Diabetes	50 μg vaginally 50 μg orally Every 6 h Maximum of 8 doses	167 paricipants 7 excluded 160 completed the study	Interval from induction to delivery
Shetty (2001) [40]	1. Parity < 5 2. Gestational age 37–42 w 3. Singleton pregnancy 4. Cephalic presentation 5. Normal FHR tracing 6. Bishop score < 8	1. Previous uterine surgery 2. Significant fetal or medical concerns 3. Contraindications to use of PG	N/A	50 μg vaginally 50 μg orally Every 4 h Maximum of 5 doses	245 included	Interval from induction to vaginal delivery
How (2001) [65]	1. Gestational age 32–42 w 2. Singleton pregnancy 3. Cephalic presentation 4. Normal FHR tracing 5. Bishop score ≤ 6	1. Nonreassuring FHR 2. Contraindications to normal labour 3. More than 1 low transverse uterine scar 4. Allergy to prostaglandins	1. Obstetric indications 2. Non-obstetric indications	25 μg vaginally and 25 μg orally 25 μg vaginally and placebo orally Placebo vaginally and 25 μg orally Every 4 h	110 participants in each arm 1 excluded	Frequence of successful induction
Wing (2000) [43]			1. Oligohydramnios 2. Preeclampsia] 3. PROM 4. Diabetes mellitus 5. Cholestasis of pregnancy 6. Potdate 7. Rh isoimmunization 8. Abnormal FHR tracing 9. Macrosomia 10. IUGR 11. Chronic hypertension 12. Other	25 μg vaginally 100 μg orally Every 4 h Maximum of 6 doses	236 participants 2 excluded 234 included	Frequence of successful induction (vaginal delivery within 24 h of induction)
Wing 1999	1. Single live fetus 2. Cephalic presentation 3. Reactive fetal heart rate pattern 4. Maternal age of ≥ 18 y	1.Contraindications for the use of PG analogues 2. Previous uterine scar 3. Bishop score ≥ 5 4. Uterine contractions > 8/h 5. Estimated fetal weight ≥ 4500 g or < 2000 g 6. Chorioamnionitis 7. Cephalopelvic disproportion 8. Parity of ≥ 6 9. Ruptured membranes 10. Placental previa or unexplained vaginal bleeding 11. Active herpes simplex Evidence or other infection 12. Moderate or severe preexisting medical disease	1. Prolonged pregnancy 2. Intrauterine fetal growth restriction 3. Oligohydramnios 4. Gestational hypertension 5. Gestational diabetes 6. Macrosomia 7. Abnormal fetal heart rate pattern 8. Cholestasis of pregnancy Breech, followed by external cephalic version 9. Isoimmunization	50 μg orally 25 μg vaginally Every 4 h, maximum of 6 doses	220 included	Compare orally administered with vaginally administered misoprostol for cervical ripening and labor induction
Bennett (1998) [18]	1. Gestational age > 37 w 2. Single live fetus 3. Cephalic presentation 4. Intact membranes	1. Contraindications for the use of PG analogues 2. Abnormal fetal heart rate 3. Previous uterine scar 4. Age < 18 years 5. Contraindications to vaginal birth	1. Prolonged pregnancy 2. Oligohydramnios	50 μg orally 50 μg vaginally Every 4 h	206 included	Test the null hypothesis that administering misoprostol orally or vaginally will result in no difference in time to vaginal birth, and to determine whether different frequencies of tachysystole and hyperstimulation are associated with route of administration
Adair (1998) [42]	1.Bishop score = < 6 2.Single live fetus 3.Gestational age ≥ 24 4.Absence of uterine contractions 5.Intact membranes	1.Placenta previa 2.Fetal malpresentation 3.Prolapsed umbilical cord 4.Previous uterine incision 5.Active genital herpes	1.Oligohydramnios 2.Preeclampsia 3.Postdates 4.Abnormal FHR tracing 5.Diabetes 6.Other	50 μg vaginally 200μ orally Every 6 h Maximum of 3 doses	178 included	Vaginal birth interval
Toppozada (1997) [54]	1. Gravida 1–4 2. Parity 0–3 3. Gestational age 37–42w 4. Single live fetus 5. Cephalic presentation 6. Bishop score = < 4 7. No contraindications for labor induction or PG administration	N/A	1. Diabetes 2. Pregnancy induced hypertension 3. Postdates	100μ vaginally, repeat after 3 h, double 3rd dose (200 μg) if needed (maximoum 100 μg) 100 μg orally, double (200 μg) 2nd dose if needed Every 3 h	40 included	Evaluate the effect of treatment to bishop score and determine the safety and success of delivery by either route of administration

**Table 5:** Patients’ characteristics

Author Year	Maternal age (years)	Gestational age at delivery (weeks)	Parity	Birth weight (g)	Bishop score	BMI (kg/m^2^)
Vaginal Administration vs Per os Administration						
Mehta (2020) [27]	25 (20–40) vs 26 (20–40)	39 (37–42) vs 39 (37–42)	68% primigravida 32% multigravida vs 66% primigravida 34% multigravida	N/A vs N/A	4.0 (3.5–4.5) vs 3.5 (3.0–4.0	N/A vs N/A
DebBarma (2020) [70]	18–30 Vs 18–30	> 37 Vs > 37	N/A Vs N/A	N/A Vs N/A	< 5 Vs < 5	N/A Vs N/A
Wallstrom (2019) [33]	32.9 ± 4.9 vs 32.3 ± 5.0	40.59 ± 1.4 vs 284.7 ± 8.8	100% primiparous vs 100% primiparous	3670.2 ± 439.8 vs 3526.9 ± 474.3	2.7 ± 1.3 vs 2.4 ± 1.3	24.4 ± 4.4 vs 24.0 ± 3.5
Hokkila (2019) [23]	30.7 ± 5.6 vs 30.0 ± 6.0	40.4 ± 3 vs 41.6 ± 2	100% nulliparous vs 100% nulliparous	3624 ± 407 vs 3706 ± 462	3 ± 2 vs 3 ± 2	25.4 vs 25.0
Sharma (2019) [44]	24.05 ± 2.88 vs 25.10 ± 3.4	40.07 ± 1.00 vs 39.81 ± 1.06	68 primigravida 32 multigravida Vs 65 primigravida 35 multigravida	N/A vs N/A	4.06 ± 1.35 Vs 3.98 ± 1.55	N/A Vs N/A
Young (2020) [35]	28.8 ± 5.6 vs 29.1 ± 6.6	39.4 ± 1.4 vs 40.0 ± 1.5	0.5 ± 0.8 vs 0.4 ± 0.6	3621 ± 557 vs 3574 ± 523	4.1 ± 1.9 vs 3.8 ± 1.9	33.9 vs 33.7
Galidevara (2018) [21]	24.7 ± 3.8 vs 24.4 ± 3.4	38.2 ± 1.8 vs 38.3 ± 1.7	60.2% primiparous 39.8% multiparous vs 63.8% primiparous 36.2% multiparous	N/A vs N/A	(2) 0% (3) 13.3% (4 ) 61.4% (5) 25.3% vs (2) 2.5% (3) 8.8% (4) 60% (5) 28.7%	N/A vs N/A
Prameela (2018) [57]	N/A vs N/A	N/A vs N/A	N/A vs N/A	N/A vs N/A	N/A vs N/A	N/A vs N/A
Madhu (2017)	22.80 ± 3.32 vs 22.06 ± 2.469	N/A VS N/A	72% primigravida vs 70% primigravida	N/A vs N/A	1–2: 18 vs 19 3–4: 31 vs 30 5–6: 1 vs 1	N/A vs N/A
Ezechukwu (2015) [56]	28.24 ± 3.66 vs 27.24 ± 4.53	40.66 ± 1.63 vs 40.59 ± 1.48	60% nulliparous 15.7% primiparous 24.3% multiparous vs 62.9% nulliparous 15.7% primiparous 21.4% multiparous	3320 ± 510 Vs 3440 ± 420	0–5: 55.7% vs 61.4% 6: 1344.3% vs 38.6%	N/A vs N/A
Deshmukh (2013) [45]	21.36 ± 2.048 vs 21.64 ± 2.342	39.52 ± 1.8785 vs 39.44 ± 1.902	Primigravida vs primigravida	2730 ± 447 vs 2820 ± 377	3.17 ± 0.7218 vs 3.09 ± 0.6921	N/A vs N/A
Komala (2013) [38]	N/A vs N/A	N/A vs N/A	62 primigravida 38 multipara Vs 64 primigravida 36 multipara	N/A vs N/A	N/A vs N/A	N/A vs N/A
Souza (2013) [37]	23.5 ± 6.7 vs 23.0 ± 6.1	39.5 ± 1.4 vs 39.3 ± 1.3	Nulliparity 64% vs Nulliparity 73%	3189.9 ± 463.2 vs 3181.2 ± 365.1	4 (3–5) vs 4 (3–6)	N/A vs N/A
Rahman (2013) [48]	N/A vs N/A	N/A vs N/A	Primigravida 62 multipara 38 Vs Primigravida 64 Multipara 36	N/A vs N/A	N/A vs N/A	N/A vs N/A
Jindal (2011) [52]	25 vs 26	39 vs 39	46.16% primigravida vs primigravida 62.75%	2800 vs 2890	N/A vs N/A	N/A vs N/A
Mehrotra (2010) [47]	26.24 ± 4.84 vs 25.22 ± 4.98	37.8 ± 1.9 vs 38 ± 1.6	41 nulliparous 27 multiparous vs 26 nulliparous 34 multiparous	N/A vs N/A	3.4 ± 1.1 vs 3.3 ± 1.0	N/A vs N/A
Ayaz (2009) [39]	35.9 vs 34.3	N/A vs N/A	2.4 ± 0.9 vs 2.9 ± 1.1	3073 ± 390 vs 2965 ± 430	N/A vs N/A	N/A vs N/A
Sheikher (2009) [69]	N/A	N/A	N/A	N/A	N/A	N/A
Cheng (2008) [63]	29.5 ± 3.9 vs 28.3 ± 4.6	40 vs 39.1	76 nulliparous 30 multiparous vs 44 nulliparous 57 multiparous	3100 vs 3050	≤ 3: 33 vs 36	N/A vs N/A
Elhassan (2007) [46]	N/A vs N/A	N/A vs N/A	32% primigravida vs 32% primigravida	3050 vs 3160	N/A vs N/A	N/A vs N/A
Rasheed (2007) [29]	23.99 ± 4.03 vs 24.76 ± 4.48	40.86 ± 3.84 vs 41.07 ± 4.45	0.73 ± 1.14 vs 0.98 ± 1.48	3008 ± 0.48 vs 3.016 ± 0.47	4.63 ± 2.18 vs 4.52 ± 2.03	N/A vs N/A
Rizvi (2007) [67]	27.8 ± 5.8 vs 28.8 ± 6.6	38.4 ± 1.9 vs 38.7 ± 1.8	1.4 ± 1.6 vs 1.4 ± 1.5	N/A	N/A	N/A
Sultana (2006) [55]	22.34 (3.16) vs 23.26 (0.418)	40.19 (1.38) vs 39.93 (1.42)	35 nulliparous 15 multiparous vs 27 nulliparous 23 multiparous	2880 vs 2920	1.8(1.12) vs 2.1(1.23)	N/A vs N/A
Adam (2005) [49]	N/A vs N/A	N/A vs N/A	N/A vs N/A	3040 ± 450 vs 2970 ± 380	N/A vs N/A	N/A vs N/A
Colon (2005) [50]	27.2 ± 6.4 vs 28.1 ± 6.7	39.1 ± 1.8 vs 38.8 ± 1.9	85 nulliparous 26 multiparous vs 56 nulliparous 37 multiparous	3352 ± 547 vs 3283 ± 610	= < 2: 70 vs 48	N/A vs N/A
Paisarntantiwong (2005) [59]	25.3(5.8) vs 25.6(5.7)	41(1.0) vs 41.3(0.4)	44 nulliparous 29 multiparous vs 45 nulliparous 28 multiparous	3200(2500–4300) vs 3300(2270–4320)	3(0–6) vs 3(0–6)	28.6(4.4) vs 28.3(3.6)
Pongsatha (2005) [61]	N/A vs N/A	N/A vs N/A	N/A vs N/A	2724 ± 515.1 vs 2737.7 ± 55	N/A vs N/A	N/A vs N/A
Uludag (2005) [62]	26.2 ± 5.2 vs 28.0 ± 5.5	38.8 ± 2.6 vs 39.7 ± 1.9	1.7 ± 1.1 vs 0.6 ± 0.7	3090 ± 659 vs 2380 ± 557	3.3 ± 1.5 vs 3.3 ± 1.2	N/A vs N/A
Paungmora-(2004) [28]	28.2 ± 4.7 vs 29.1 ± 4.9	40.5 (37–41) vs 41 (37- 42)	Nulliparity 73.7% vs Nulliparity 78.7%	3211.4 ± 53.1 vs 3207.9 ± 47.7	3 (0–6) vs 3 (0–6)	23.3 vs 24.1
Schneider (2004) (congress abstract) [68]	N/A	N/A	N/A	N/A	N/A	N/A
Nopdonrattakoon (2003) [53]	25.3 ± 5.5 vs 24.9 ± 5.5	39.1 ± 1.1 vs 39.0 ± 1.0	Gravida: 1: 22 vs 24 2: 23 vs 16 3: 5 vs 8 4: 3 vs 5	3051.3 ± 322.6 vs 3001.9 ± 288.9	4 (2.4) vs 4 (2.4)	223.5 ± 2.0 vs 23.8 ± 2.3
Shetty (2003) [41]	28 (5.5) vs 28.6 (6.2)	286 (7.7) days vs 285 (8.9) days	28 primigravida vs 29 primigravida	3695 (773) vs 3621 (474)	4 (2.2) vs 4.1 (1.7)	N/A vs N/A
Hall (2002) [58]	N/A vs N/A	N/A vs N/A	36 nulliparous 12 multiparous vs 33 nulliparous 26 multiparous	3281 ± 507 vs 3359 ± 541	N/A vs N/A	N/A vs N/A
Le Roux (2002) [66]	27.9 vs 28.1	39 vs 38.3	52 nulliparous vs 43 nulliparous	NA	4 vs 3	N/A
Carlan (2001) [60]	25.8 ± 6.5 vs 25.4 ± 6.4	37.6 ± 2.8 vs 37.8 ± 2.6	203 primigravida 290 nulliparous Vs 231 primigravida 308 nulliparous	2959 ± 735 vs 2992 ± 706	3 vs 3	N/A vs N/A
Fisher (2001) [64]	27 (19–37) vs 27 (17–41)	41 (33–42) vs 41 (36–42)	36 nulliparous vs 40 nulliparous	N/A vs N/A	3 (0–8) vs 3 (0–6)	N/A vs N/A
Kwon (2001) [51]	27.6 (5.1) vs 27.2 (5.4)	40.3 (1.7) vs 40.3 (1.8)	43 nulliparous vs 44 nulliparous	3749 (562) vs 3526 (490)	N/A vs N/A	N/A vs N/A
How (2001) [65]	23.5 ± 6.1 vs 23.4 ± 5.9	38.8 ± 3.1 vs 39.0 ± 2.0	53 nulliparous vs 56 nulliparous	3133 ± 633 vs 3149 ± 662	3.5 ± 1.6 vs 3.8 ± 0.7	NA
Shetty (2001) [40]	28 (15–46) vs 28 (16–43)	41 (37–42) vs 41 (37–42)	76 primigravida vs 73 primigravida	3679 (474) vs 3625 (519)	3 (0–6) Vs 4 (0–7)	N/A vs N/A
Wing (2000) [43]	28.8 ± 6.6 vs 27.8 ± 5.8	38.7 ± 1.8 vs 38.4 ± 1.9	1.4 ± 1.5 vs 1.4 ± 1.6	3179 ± 570 vs 3196 ± 454	2 (0–7) vs 2 (0–7)	N/A vs N/A
Wing 1999	N/A vs N/A	38.6 ± 2.0 vs 39.2 ± 1.7	Nulliparity 48.2% vs Nulliparity 48.2%	3272.0 ± 584.6 vs 3264.5 ± 531.2	2 vs 2	N/A vs N/A
Adair (1998) [42]	24.5 ± 6.7 vs 24.4 ± 6.0	38.3 ± 2.5 vs 37.8 ± 3.4	36 primigravida 49 nulliparous Vs 37 primigravida 47 nulliparous	2869 ± 709 vs 2715 ± 720	N/A vs N/A	N/A vs N/A
Bennett (1998) [18]	28.7 ± 4.9 vs 27.5 ± 5.0	40.6 ± 1.23 vs 40.77 ± 1.08	Nulliparity 72.6% 0.3 ± 0.6 vs Nulliparity 66.3% 0.5 ± 0.8	3645 ± 566 vs 3585 ± 612	< 7 79.4% vs < 7 76%	N/A vs N/A
Toppozada (1997) [54]	27.5 ± 4.51 vs 29.15 ± 5.4	40.3 ± 1.87 vs 40.85 ± 1.57	0.8 ± 0.95 vs 1.25 ± 1.16	N/A vs N/A	2.25 ± 1.69 vs 1.85 ± 1.39	N/A vs N/A
Vaginal Administration vs Sublingual Administration						
Dadashaliha (2021) [20]	27.7 ± 5.1 vs 28.1 ± 4.7	39.1 ± 1.8 Vs 39.1 ± 1.9	34.9% primiparous 31.2% multiparous vs 34.9% primiparous 31.2% multiparous	2200 ± 560 vs 2200 ± 620	0.92 ± 0.76 Vs 0.90 ± 0.83	24.03 ± 3.7 Vs 24.4 ± 3.4
Gattás 2018 [22]	25.9 ± 6.4 vs 26.5 ± 6.9	39.1 ± 1.5 vs 38.7 ± 1.4	0 (0–1) vs 0 (0–1)	3322.1 ± 426.9 vs 3268.3 ± 458.9	3 (2–4) vs 3 (2–4)	N/A vs N/A
Ifariola (2020) [24]	29.3 ± 3.9 vs 29.4 ± 3.5	N/A vs N/A	N/A vs N/A	3180 ± 410 vs 3100 ± 310	2.56 ± 0.95 vs 2.28 ± 0.88	N/A vs N/A
Agrawal (2020) [15]	N/A vs N/A	N/A vs N/A	N/A vs N/A	N/A vs N/A	N/A vs N/A	N/A vs N/A
Bansal (2019) [16]f	23.55 ± 3.68 vs 23.47 ± 3.32	40.33 ± 2.14 vs 39.95 ± 2.04	1.54 ± 0.23 vs 1.36 ± 0.79	N/A vs N/A	3.79 ± 0.83 vs 3.66 ± 1.1	N/A vs N/A
Sheir (2019) [32]	25.21 ± 3.86 vs 25.85 ± 4.27	39.66 ± 1.83 vs 39.20 ± 1.71	P1 28.8% P2 25.0% PG 46.2% vs P1 30.8% P2 32.7% PG 36.5%	N/A vs N/A	2.83 ± 0.79 vs 3.00 ± 1.08	N/A vs N/A
Khan (2018) [26]	25.24 ± 3.17 vs 25.14 ± 2.9	39.39 ± 1.18 vs 39.44 ± 1.13	N/A vs N/A	N/A vs N/A	4.64 ± 1.35 vs 5.02 ± 1.27	N/A vs N/A
Galidevara (2018) [21]	24.7 ± 3.8 vs 24.4 ± 3.4	38.2 ± 1.8 vs 38.3 ± 1.7	60.2% primiparous 39.8% multiparous vs 63.8% primiparous 36.2% multiparous	N/A vs N/A	(2) 0% (3) 13.3% (4) 61.4% (5) 25.3% vs (2) 2.5% (3) 8.8% (4) 60% (5) 28.7%	N/A vs N/A
Jahromi (2016) [25]	26.59 ± 4.63 vs 25.94 ± 4.21	284.36 ± 7.69 vs 284.86 ± 6.50	100% primiparous vs 100% primiparous	3291.50 ± 257.42 vs 3287.70 ± 268.79	4.78 ± 1.54 vs 4.84 ± 1.50	N/A vs N/A
Sheela (2015) [31]	23.86 ± 3.553 vs 24.20 ± 3.836	39.34 ± 0.855 vs 39.30 ± 1.096	Primi 58% Multi 42% vs Primi 67% Multi 33%	N/A vs N/A	3.90 ± 0.482 vs 3.93 ± 0.573	N/A vs N/A
Sharami (2014) [30]	23.5 ± 4.3 vs 25 ± 4.6	39 ± 1 vs 39 ± 0.8	100% primiparous vs 100% primiparous	3268 ± 314 vs 3237 ± 389	2.1 ± 0.7 vs 2.3 ± 0.6	N/A vs N/A
Zahran (2009) [36]	26.7 ± 2.8 vs 28.2 ± 2.5	40.7 ± 1.8 vs 40.5 ± 2.0	2.4 ± 1.1 vs 2.2 ± 0.9	N/A vs N/A	2.4 ± 1.2 vs 2.2 ± 1.2	N/A Vs N/A
Elhassan (2007) [46]	N/A vs N/A	N/A vs N/A	32% primigravida vs 30.8% primigravida	3050 vs 3140	N/A vs N/A	N/A vsN/A
Bartusevicius (2006) [17]	29 ± 5.3 vs 27 ± 5.2	40 ± 1.1 vs 41 ± 0.9	1.5 ± 0.7 vs 1.5 ± 0.8	3549.4 ± 361.9 vs 3568.3 ± 371.6	4.1 ± 1.0 vs 4.1 ± 1.0	24 ± 5.9 vs 25 ± 5.8
Caliskan (2005) [19]	27.2 ± 5.4 vs 27.5 ± 5.3	39.86 ± 1.57 vs 39.43 ± 1.43	Nulliparity 52.5% vs Nulliparity 47%	3341 ± 534 vs 3,298 ± 514	1.7 ± 1 vs 1.9 ± 1.1	28.1 ± 4.8 vs 27.9 ± 4.6
